# Potential application of spectral indices for olive water status assessment in (semi‐)arid regions: A case study in Khuzestan Province, Iran

**DOI:** 10.1002/pld3.494

**Published:** 2023-06-06

**Authors:** Azimeh Asgari, Abdolrahim Hooshmand, Saeed Broumand‐Nasab, Shohreh Zivdar

**Affiliations:** ^1^ Irrigation and Drainage, Water and Environmental Engineering College Shahid Chamran University of Ahvaz Ahvaz Iran; ^2^ Horticulture, College of Agriculture Shahid Chamran University of Ahvaz Ahvaz Iran

**Keywords:** olive, plant water status, relative water content, soil water content, spectral indices, water stress

## Abstract

Spectral indices can be used as fast and non‐destructive indicators of plant water status or stress. It is the objective of the present study to evaluate the feasibility of using several spectral indices including water index (WI) and normalized spectral water indices 1–5 (NWI 1–5) to estimate water status in olive trees in arid regions in Iran. The experimental treatments involved two olive cultivars (Koroneiki and T2) and four irrigation regimes (irrigated with 100%, 85%, 70%, and 55% estimated crop evapotranspiration [ETc]). The results obtained showed that olive trees subjected to the different irrigation regimes of 85%, 70%, and 55% ETc experienced soil water content (SWC) deficits by 4.5%, 12%, and 20.5% that of the control, respectively. Significant differences were observed among the treatments with respect to measured relative water content (RWC), SWC, and the spectral indices of WI and NWI 1–5. The normalized spectral indices combining NIR and NIR wavelengths were found more effective in tracking changes in RWC and SWC than those that combine NIR and VIS or VIS and VIS wavelengths, respectively. Spectral indices were closely and significantly associated with RWC ($.63^{**}<R^{2}<.77^{**}$) and SWC ($.51^{**}<R^{2}<.67^{**}$). Among all the spectral indices investigated, NWI‐2 showed the least consistent associations with RWC (ranging from 4–15% lower than the other indices examined) and SWC (ranging from 1–23% lower than the others). Based on the pooled data on spectral indices, RWC, and SWC collected during the study period, WI, NWI‐1, NWI‐4, and NWI‐5 showed stronger correlations with RWC and SWC than did NWI‐3 and NWI‐2. In conclusion, the spectral indices of WI and NWI 1–5 measured at the leaf level are found useful as fast and non‐destructive estimators of plant water stress in arid regions.

## 1 INTRODUCTION

The olive tree (*Olea europaea* L.) is a perennial evergreen tree native to the Mediterranean regions (Arenas‐Castro et al., 2020) with a high degree of resistance to aridity (Connor & Fereres, 2005). It is a most important tree crop worldwide in terms of the area under cultivation amounting to almost 11 million hectares (Garcia‐Tejero et al., 2017). While oil extraction accounts for about 90% of the world's olive production, the remaining 10% is used as table olive (FAOSTAT, 2018). The growing concern for human longevity and health alongside the rising public awareness of olive oil's nutritional value has increased its consumption so that the area under its cultivation has witnessed a rapid growth over the past few decades (IOC, 2018) and its production and consumption will expectedly continue to rise in future (Fabbri, 2004). Being no exception, Iran has also witnessed a growth in both area and geographic distribution of olive cultivation.

It is common knowledge that irrigated agriculture is facing a rising challenge in terms of increasing water deficit and uncertainty in water supplies not only because of the prolonged droughts and the global climate change but also due to the escalating competition among environmental, domestic, and industrial water demands (DeJonge et al., 2015). These considerations motivate maximum water productivity in all agricultural activities including olive production.

One option to consider in this situation is deficit irrigation whereby irrigation demand is reduced and crop water use efficiency is enhanced (Mairech et al., 2020). It has also been indicated that optimization of olive oil quantity and quality requires finely tuned water management as increased irrigation, up to a certain level, might result in higher yields but a certain degree of stress is concurrently known to improve oil quality (Ben‐Gal, Dag, et al., 2011; Ben‐Gal, Yermiyahu, et al., 2011; Berenguer et al., 2006; Dag et al., 2008). However, achieving this fine balance between water use and crop yield requires both a comprehensive knowledge of crop response to water stress and an optimized irrigation schedule (Geerts & Raes, 2009).

Optimization of irrigation relies on identification of real time crop conditions and its sensitivity to water stress, both being reflections of specific physiological status, soil moisture, and climatic conditions (Centritto et al., 2000; Tognetti et al., 2009). It is, therefore, beneficial to exploit monitoring tools that provide accurate information regarding crop water status.

Crop water status may be determined by soil‐based measurements, direct sensing of plant water status parameters, or indirect sensing of plant response to stress. Soil‐based assessments include point measurements of water content and/or water potential; these are limited due to the difficulty and expense of satisfactorily representing the heterogenic conditions found in the root zone (Campbell & Campbell, 1982; Charlesworth, 2005). Plant water stress may be measured in terms of stem water potential; stomatal conductance; sap‐flow; or as changes in leaf, stem, or trunk size; canopy water content (CWC); and relative water content (RWC). While all of these techniques are capable of providing accurate reports of actual crop water stress, they are destructive, labor intensive, localized, limited by small sample size, and unsuitable for automation (Ballester et al., 2013; Berni et al., 2009; Cohen et al., 2005; Gontia & Tiwari, 2008; Jones, 1999; Leinonen & Jones, 2004). It is, therefore, instructive to develop reliable, fast, simple, practical, and economical high‐throughput sensing methods for the assessment of water stress in crops (Elsayed et al., 2017).

Spectral reflectance techniques have been demonstrated to be instantaneous, cheap, and non‐destructive alternative methods for integrative large‐scale assessment of several phenotypic parameters under different environmental conditions (El‐Hendawy, Al‐Suhaibani, et al., 2017; El‐Hendawy, Hassan, et al., 2017; Elsayed et al., 2017; Gitelson et al., 2003; Lobos et al., 2014; Rapaport et al., 2017; Serrano et al., 2010; Sun et al., 2008). An additional advantage of reflectance indices is that they can be used for assessing plant water status since they change in response to crop water content (Penuelas et al., 1997; Stimson et al., 2005; Ustin et al., 1998). For instance, drought stress influences spectral reflectance values by causing alterations in leaf cell structure and composition via such changes in the relationships between cell walls and air spaces, cell sizes and shapes, and/or cell wall composition and structure (Grant, 1987; Penuelas et al., 1994).

Previous study has shown that a number of spectral indices might be used to estimate plant water status (El‐Shikha et al., 2007; Gutierrez et al., 2010; Schmidhalter et al., 2001; Yang et al., 2016). These include the normalized spectral water indices of NWI‐1: (R970 − R900)/(R970 + R900), NWI‐3: (R970 − R920)/(R970 + R920), NWI‐4: (R970 − R880)/(R970 + R880), and NWI‐5: (R970 − R910)/(R970 + R910) as well as those based on 992 and 900 nm (viz., NWI992‐900; (R992 − R900)/(R992 + R900)) obtained from passive reflectance sensor measurements that can explain variations in plant water content, RWC, and grain yield of wheat cultivars subjected to different irrigation regimes (Elsayed et al., 2017). Marino et al. (2014) reported that the water index (WI): R970/R900, the photochemical reflectance index PRI: (R531 − R570)/(R531 + R570), and the normalized difference vegetation index NDVI: (R800 − R680)/(R800 + R680) were capable of being effectively used to assess the effects of drought on olive trees. Moreover, Gutierrez et al. (2010) observed that the normalized spectral water indices of NWI‐1: (R970 − R900)/(R970 + R900), NWI‐2: (R970 − R850)/(R970 + R850), NWI‐3: (R970 − R920)/(R970 + R920), and NWI‐4: (R970 − R880)/(R970 + R880) demonstrated a great potential in differentiating high‐ and low‐yielding genotypes in advanced lines of spring wheat under well‐irrigated, water‐stressed, and high‐temperature conditions in diverse trials. In bell pepper plants, the photochemical reflectance indices centered at 553 nm, PRI553: (R553 − R531)/(R553 + R531); WI; the renormalized difference vegetation index RDVI: (R800 − R670)/(R800 + R670)^1/2^; the normalized photochemical reflectance index of PRInorm: PRI/(RDVI (R700/R670)); and the ratio of WI to normalized difference vegetation index (WI/NDVI) have been reportedly the most useful indices for detecting water stress (Ihuoma & Madramootoo, 2019). Stimson et al. (2005) found that the normalized difference water index of NDWI: (R860 − R1240)/(R860 + R1240) and the normalized difference vegetation index of NDVI: (R900 − R680)/(R900 + R680) exhibited significant correlations with leaf water content and water potential in two conifer species (viz., *Pinus edulis* and *Juniperus monosperma*). Zarco‐Tejada and Ustin (2001) and Zarco‐Tejada et al. (2003) modeled the simple ratio of WI (SRWI: R860/R1240) to estimate vegetation water content based on leaf thickness, biomass, and leaf area index.

Although a large number of indices at diverse wavelengths have been proposed based on theoretical considerations for assessing plant water status, they have not been adequately validated against field data (Serrano et al., 2000; Sims & Gamon, 2003). It is the objective of the present study to determine the performance of select spectral indices in assessing such water status related parameters in olive cultivars as RWC and soil water content (SWC) under different irrigation regimes in the arid region of Ahvaz, Iran.

## 2 MATERIALS AND METHODS

### 2.1 Field experiment and design

The experiment was performed in the research orchard of the Department of Horticultural Science, Shahid Chamran University of Ahvaz, Khuzestan Province, Iran (Latitude: 31°20′N, Longitude: 48°40′E, Elevation: 22.5 m a.s.l) during the irrigation season of 2018 from May 22 to December 21. The region is characterized by a (semi‐)arid climate with an average annual precipitation of 164.4 mm and an annual evaporation from open pans of 3092.6 mm calculated for the 2008–2017 period using the data recorded at a standard weather station at Ahvaz International Airport where maximum air temperature might exceed 50°C during the hottest months of July and August but rarely ever drops below 0°C during the cold months of December and January. Most rainfall events in the region occur from November to February, with dry and hot spring and summer months.

The orchard used in this study housed mature (17‐year‐old) olive (*O. europaea* L.) trees of the two Koroneiki and T_2_ cultivars planted at spaces of 5 × 6 m. The trees were irrigated every 3 days using a drip irrigation system with a nominal factory discharge of 75 L h^−1^, 1.5–7 bar, Pressure Compensating (Iran Drip, Iran) supplying water through a pipeline running between the tree rows and one bubbler for each tree. The experiments were conducted using four irrigation regimes, namely, IR_1_, irrigated up to 100% estimated crop evapotranspiration (ETc); IR_2_, irrigated up to 85% ETc; IR_3_, irrigated up to 70% ETc; and IR4, irrigated up to 55% ETc.

**FIGURE 1:**
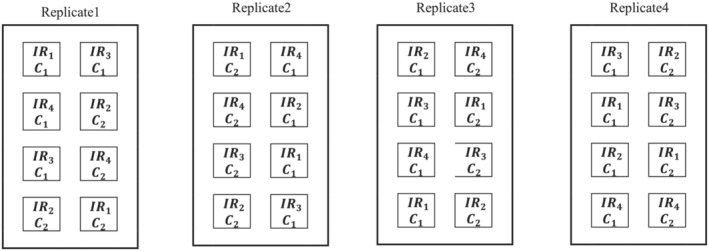
Experimental design layout of the field experiment in a split plot arrangement in a Randomized Complete Block Design (RCBD). IR_1_, irrigated up to 100% estimated crop evapotranspiration (ETc); IR_2_, irrigated up to 85% ETc; IR_3_, irrigated up to 70% ETc; and IR_4_, irrigated up to 55% ETc. C_1_, cultivar 1 (Koroneiki); C_2_, cultivar 2 (T_2_).

The split plot was used as the experimental design arranged based on the Randomized Complete Block Design (RCBD) with four replications. The two olive cultivars were designated as the main factors and the four irrigation regimes as the sub‐main factors. A total of 32 trees were used in this study (16 trees of each cultivar). Such that four trees (one tree per block) of each cultivar per irrigation treatment could be measured (Figure 1).

The upper 1.0 m of the soil profile at the experimental site is characterized by a sandy clay loam texture containing 52% sand, 24% silt, and 23% clay with an electrical conductivity of 5.6 dS m^−1^, an organic content of 1.7%, a bulk density of 1.43 g cm^−3^, a total N of .5%, an Olsen‐P value of 30.7 mg kg^−1^, and a pH of 7.38. The SWC at field capacity (−.3 MPa) and at wilting point (−1.5 MPa) were determined to be .21 and .09, respectively.

### 2.2 Irrigation water demand

Crop water requirement (ETc) for the full irrigation regime (1.00 ETc) was determined using the following equation:

(1) ETc=ETo×Kc.

The reference daily evapotranspiration (ETo) as calculated by the Food and Agriculture Organization (FAO) according to the modified Penman–Monteith method (Allen et al., 1998) is expressed by Equation (2):

(2) $$ET_{0}=\frac{0.408\Delta (Rn-G)+\gamma \frac{900}{T+273}U_{2}(e_{s}-e_{a})}{\Delta +\gamma (1+0.34\;U_{2})}$$

where Eto is the reference evapotranspiration (mm day^−1^), Rn is the net radiation at crop surface (MJ m^−2^ day^−1^), G is the soil heat flux density (MJ m^−2^ day^−1^), T is the air temperature at 2‐m height (°C), u_2_ is the wind speed at 2‐m height (m s^−1^), es is the saturation vapor pressure (kPa), ea is the actual vapor pressure (kPa), es−ea is the saturation vapor pressure deficit (kPa), ∆ is the slope of vapor pressure curve (kPa°C^−1^), and γ is the psychrometric constant (kPa°C^−1^).

The values of crop coefficient (Kc) for olive tree suggested by FAO‐56 were adjusted to the conditions prevalent in the study area. The total amount of irrigation water applied during the study period for 1.00 ETc was 651 mm, which was subsequently reduced to 554, 456, and 328 mm in the 85%, 70%, and .55% ETc treatments, respectively.

### 2.3 Leaf sampling

The leaf sampling was accomplished on three different days on July 3, October 29, and December 16, 2018, at midday between 11.00 and 14.00 h under cloudless conditions. In overall in every single sampling, eight fully developed Sun‐exposed leaves, which were located in the middle of canopy were excised from each tree (five leaves were used for RWC measurements, and three more leaves were used for spectral measurements). The leaf samples were then sealed in plastic bags and kept at temperatures below 5°C before they were transferred to the laboratory.

### 2.4 Relative water and soil water measurements

RWC was used to describe the water status of the two olive cultivars under the four irrigation regimes. RWC was determined in accordance with the procedure set out by Turner (1981). In this procedure, leaf samples were weighed immediately after its excision, to obtain leaf fresh weight (FW) before they were rehydrated in de‐ionized water for approximately 24 h at 25°C until they grew fully turgid. Following rehydration, each leaf was blotted and immediately weighed to obtain their turgid weight (TW). Subsequently, the samples were dried in an oven at 70°C to obtain constant leaf dry weight (DW). Finally, RWC was determined as the average of the values measured in five fully expanded leaves from each tree using the following equation.

(3) $$\mathrm{RWC}\;\left(\%\right)=\left(\mathrm{FW}-\mathrm{DW}\right)/\left(\mathrm{TW}-\mathrm{DW}\right)$$

Soil water gravimetric measurements were performed simultaneously with the RWC measurements using samples taken at a depth of 20 cm and an average distance of 50 cm from the tree trunk.

### 2.5 Spectral reflectance measurements

Concurrent with other measurements, spectral reflectance was measured using a portable spectrometer (ASD FieldSpec 3, Analytical Spectral Devices Inc, USA) operated in the spectral range of 350–2,500 nm with an average spectral resolution of 3 nm (Full‐Width‐Half‐Maximum) and a sampling interval of 1.4 nm at the leaf level. The handheld FieldSpec 3 sensor consisted of two units: one linked to a diffuser to measure light radiation as a reference signal and a fiber optic unit measuring canopy reflectance (Elsayed et al., 2017; Rischbeck et al., 2016).

Prior to each spectral measurement, the device was calibrated using a white barium sulfate (BaSO4) plate to provide maximum reflectance (Labsphere Inc, North Sutton, USA) (Knighton & Bugbee, 2004).

To reduce errors associated with illumination effects, a fiber optic contact probe was pressed on the leaf surface so that it would be illuminated only by the constant light source placed inside the contact probe (Contact Probe, Analytical Spectral Devices, Boulder, CO). To minimize differences in background reflectance due to electromagnetic radiation transmitted through the leaf, a spectrally black surface was placed on the underside of the leaf. Finally, the average of spectral measurements at the level of three leaves was considered as the spectrum for each tree.

The advantage of taking spectra at the leaf level is that the likely relationships between plant water status and spectral indices are not affected by background variables or atmospheric noise. It can, thus, be assumed that variations in spectral indices solely reflect changes in leaf properties. However, it is not just plant water status that might lead to variations in leaf properties; hence, no straightforward relationship can be directly established between plant water status and spectral indices.

Using the spectral reflectance data, six indices that are effective in plant water status assessment including WI = (R900/R970) (Penuelas et al., 1994); NWI‐1 = (R970 − R900)/(R970 + R900) (Babar et al., 2006); NWI‐2 = (R970 − R850)/(R970 + R850); NWI‐3 = (R970 − R880)/(R97 0 + R880); NWI‐4 = (R970 − R920)/(R970 + R920) (Prasad et al., 2007); and NWI‐5 = (R970 − R910)/(R970 + R910) (Elsayed et al., 2017) were obtained.

### 2.6 Contour plot

SigmaPlot 10.0 software (Systat, San Jose, CA, USA) was used to draw a contour plot showing the coefficients of determination (*R* ^2^) of the relationships among RWC and SWC, on the one hand, and the narrow band normalized difference spectral indices that had been calculated using all the possible two‐band combinations in the range of 350–1,300 nm (formula: (R1 − R2)/(R1 + R2)) obtained from all the measurements.

### 2.7 Statistical analysis

The ability of six spectral indices in assessing such water status related parameters in two olive cultivars as RWC and SWC under different irrigation regimes was investigated. The experiment was set up in a split plot design arranged based on the RCBD with four replicates. The main factors consist of two olive cultivars, and four irrigation regimes were tested as the sub‐main factors. Analysis of variance (ANOVA) based on split plot design was conducted on the spectral indices, RWC and SWC using PROC GLM (General Linear Model) procedure of SAS software v.9.0 (SAS, 2001). Then, the least significant difference (LSD) test was used to compare the means and determine if they were significantly different from each other at the *p* ≤ .05 level. SigmaPlot 10.0 software (Systat, San Jose, CA, USA) was used to create graphical representation of the data. Simple linear regressions were performed not only to analyze the relationships of spectral indices with RWC and SWC but to determine coefficients of determination as well. The significance levels of the relationships of RWC and SWC with spectral indices were expressed by Pearson correlation coefficients using SPSS (vers. 23.0 for Windows, SPSS Inc, Chicago, IL, USA). The regression curves were fitted to develop empirical models based on linear relationships using dataset of 64 data that was collected in July 3 and October 29 (67% of each measured RWC and SWC) as training data, whereas the remaining 32 data that were collected in December 16 (33%) was retained as test data. The coefficients of determination, root‐mean‐square error (RMSE), and the intercept and slope of the linear regressions between observed and predicted values were calculated as measures of differences between the observed and predicted RWC and SWC values.

## 3 RESULTS AND DISCUSSION

### 3.1 Contour plot

**FIGURE 2:**
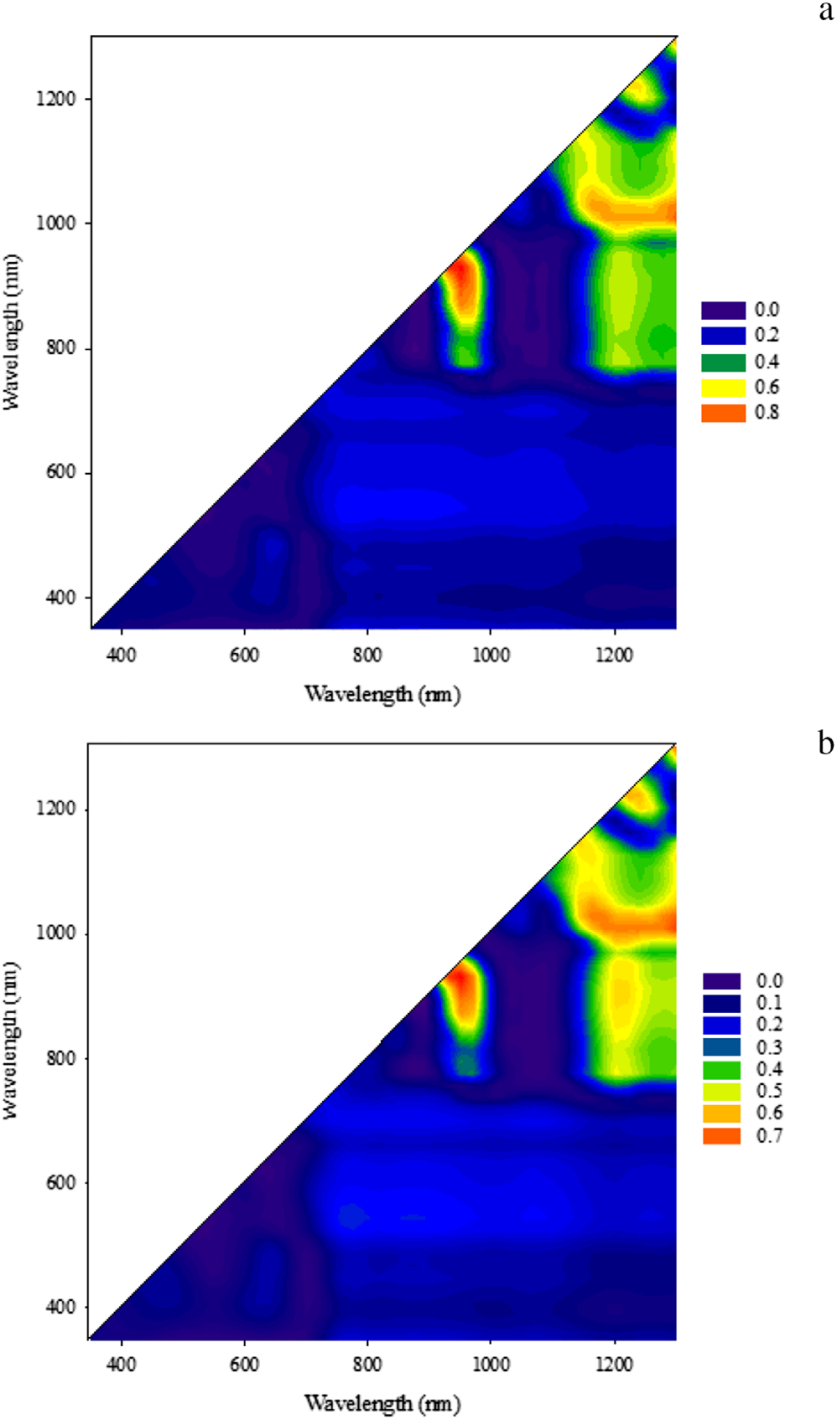
Correlation matrices (contour plots) showing the coefficients of determination (*R* ^2^) for (a) relative water content (RWC) and (b) soil water content (SWC) across the two olive cultivars studied for all the dual wavelength combinations in the 350–1300 nm range (as a normalized difference spectral index) of the hyperspectral passive reflectance sensing.

For further identification of the sensitive band regions in the visible and near‐infrared region range (350 to 1,300 nm), the coefficients of determination (*R* ^2^) obtained for the relationships of RWC and SWC with all the normalized difference spectral indices (NDSIs), obtained based on the combination of one wavelength on the horizontal axis (wavelength 1) and one on the vertical axis (wavelength 2), were plotted in contour plots that are two‐dimensional representations of three‐dimensional data. In this procedure, use was made of the pooled data from three measurements, four replications, four irrigation regimes, and two cultivars (Figure 2). Finally, linear regression fits were exploited to derive the relationships among RWC, SWC, and NDSIs.

Contour plot analysis provided mean coefficients of determination (*R* ^2^) for the relationships among the measured values as normalized differential spectral indices with RWC and SWC for all the dual wavelength combinations in the range of 350–1,300 nm. Wavelengths in the NIR region on the vertical axis exhibited significant correlations with RWC and SWC when they were combined with the wavelengths in the NIR regions on the horizontal axis. Based on *R* ^2^ values obtained, the NDSIs combining NIR and NIR wavelengths were more useful in effectively tracking changes in RWC and SWC than were those that combined NIR and VIS or VIS and VIS wavelengths. This is confirmed by El‐Hendawy et al. (2019), who reported that NDSIs based on VIS–VIS wavelengths failed to track changes in leaf water parameters and grain yield in wheat cultivars.

The contours of the spectral sensor matrices presented stronger relationships of RWC and SWC in the olive cultivars with near‐infrared wavelengths and the combination of visible and near‐infrared wavelengths than with the sole visible wavelengths.

The hotspot regions for *R* ^2^ values were confined to wavelength intervals from 800 to 1,000 nm on the vertical axis, those from 900 to 1,000 nm on the horizontal axis, those at around 1,000–1,050 nm on the vertical axis, and those around 1,100–1,300 nm on the horizontal axis. These findings agree with the results of Elsayed et al. (2017) and Gutierrez et al. (2010), who reported that the dual near‐infrared wavelength combinations as NDSIs from 750–1,000 nm and 880–970 nm were strongly influenced by such plant water status parameters as RWC and leaf water potential (LWP) in wheat cultivars. The authors ascribed this to the greater effect of wavelengths from 850 to 1,000 nm on plant water status.

### 3.2 Variations of RWC, SWC, and spectral indices between irrigation regime and cultivar

The different irrigation regimes used in this study led to varying degrees of plant water stress. The differences in effects of irrigation regime, cultivar, and their interactions on RWC, SWC, and spectral indices were calculated using the values obtained from three measurements. All the parameters involved in both RWC and SWC were found significantly affected by irrigation regime, with the RWC values obtained under the .85 ETc treatment being similar to those obtained under the full irrigation treatment (1.0 ETc) and the SWC values obtained under the .7 ETc treatment being similar to those obtained under the .55 ETc one. When averaged over three measurements, the severe water stress treatment (i.e, .55 ETc) was, however, found to have resulted in decreases of 1.78%, 4.32%, and 5.03% in RWC and to declines of 4.54%, 12.05%, and 20.48% in SWC when compared with those observed with the .70, .85, and 1.00 ETc treatments, respectively (Table 1). Generally, the highest and lowest mean values of all the RWC and SWC measurements in the two olive cultivars were recorded at ETcs of 100% and 55%, respectively (Table 1).

**TABLE 1:** Mean values of RWC, SWC, WI, and the five normalized spectral indices obtained for the two olive cultivars subjected to the four irrigation regimes.

Measured parameters	Treatments	July 3	October 29	December 16	Mean
RWC (%)					
	ETc 100%	63.98 ± 2.34a	66.78 ± 1.30a	70.04 ± 2.97a	66.94 ± 1.66a
	ETc 85%	63.64 ± 2.32a	65.83 ± 1.54a	69.85 ± 2.28a	66.44 ± 1.67a
	ETc 70%	61.26 ± 2.90b	64.62 ± 1.20b	68.84 ± 1.93ab	64.91 ± 1.51b
	ETc 55%	59.60 ± 1.38c	63.94 ± .58b	67.71 ± 1.98b	63.57 ± .71c
SWC (%)					
	ETc 100%	15.52 ± 1.75a	19.15 ± 1.86a	22.58 ± 2.63a	19.084 ± 1.66a
	ETc 85%	14.35 ± 2.03a	16.88 ± 2.11b	20.53 ± 1.70b	17.25 ± 1.65b
	ETc 70%	12.79 ± 2.19b	15.61 ± 1.43c	19.30 ± 1.87bc	15.90 ± 1.08c
	ETc 55%	12.16 ± 1.38b	14.82 ± 1.26c	18.59 ± 1.26c	15.18 ± .68c
WI					
	ETc 100%	1.0399 ± .0038a	1.0428 ± .0029a	1.0447 ± .0046a	1.0425 ± .0026a
	ETc 85%	1.0364 ± .0038b	1.0402 ± .0041ab	1.0436 ± .0055a	1.0407 ± .0027b
	ETc 70%	1.0338 ± .0036bc	1.0384 ± .0030bc	1.0425 ± .0030a	1.0382 ± .0023c
	ETc 55%	1.0327 ± .0024c	1.0359 ± .0024c	1.0409 ± .0032a	1.0366 ± .0013 cd
NWI‐1					
	ETc 100%	−.0196 ± .0018c	−.0210 ± .0014c	−.0219 ± .0022a	−.0208 ± .0012d
	ETc 85%	−.0179 ± .0018b	−.0197 ± .0020bc	−.0213 ± .0026a	−.0196 ± .0013c
	ETc 70%	−.0166 ± .0017ab	−.0188 ± .0014ab	−.0208 ± .0014a	−.0188 ± .0011b
	ETc 55%	−.0161 ± .0012a	−.0178 ± .0012a	−.0200 ± .0015a	−.0179 ± .0006a
NWI‐2					
	ETc 100%	−.0195 ± .0022b	−.0214 ± .0015c	−.0209 ± .0024a	−.0206 ± .0014c
	ETc 85%	−.0176 ± .0026a	−.0200 ± .0021bc	−.0211 ± .0031a	−.0196 ± .0014b
	ETc 70%	−.0162 ± .0021a	−.0191 ± .0018ab	−.0201 ± .0020a	−.0185 ± .0013a
	ETc 55%	−.0162 ± .0011a	−.0177 ± .0014a	−.0200 ± .0018a	−.0180 ± .0008a
NWI‐3					
	ETc 100%	−.0200 ± .0020c	−.0216 ± .0014c	−.0220 ± .0023a	−.0212 ± .0013c
	ETc 85%	−.0182 ± .0020b	−.0202 ± .0021bc	−.0218 ± .0028a	−.0207b ± .0013
	ETc 70%	−.0169 ± .0019ab	−.0194 ± .0016ab	−.0211 ± .0016a	−.0191 ± .0012a
	ETc 55%	−.0165 ± .0011a	−.0181 ± .0012a	−.0205 ± .0016a	−.0184 ± .0007a
NWI‐4					
	ETc 100%	−.0183 ± .0018c	−.0194 ± .0013c	−.0205 ± .0021a	−.0194 ± .0012d
	ETc 85%	−.0167 ± .0017b	−.0183 ± .0018bc	−.0198 ± .0022a	−.0183 ± .0011c
	ETc 70%	−.0156 ± .0017ab	−.0175 ± .0013ab	−.0194 ± .0012a	−.0175 ± .0010ab
	ETc 55%	−.0150 ± .0011a	−.0165 ± .0010a	−.0186 ± .0014a	−.0167 ± .0005a
NWI‐5					
	ETc 100%	−.0191 ± .0018c	−.0204 ± .0013c	−.0215 ± .0022a	−.0204 ± .0012d
	ETc 85%	−.0175 ± .0018b	−.0192 ± .0019bc	−.0208 ± .0024a	−.0192 ± .0012c
	ETc 70%	−.0163 ± .0017ab	−.0183 ± .0014ab	−.0204 ± .0013a	−.0183 ± .0010b
	ETc 55%	−.0157 ± .0012a	−.0172 ± .0011a	−.0196 ± .0015a	−.0175 ± .0006a

*Note*: Data are mean of four blocks per treatment ± standard deviation. Values with the same letter are not statistically different (*p* ≤ .05) across the treatments.

Elsayed et al. (2017) reported a significant decrease in RWC values of wheat plants from 78 for their control plants to 59–61% for their 50% stressed plants. Moreover, significantly reduced RWC values from 88% to 45% have also been reported during wheat development as a result of water stress (Siddique et al., 2000). Sun et al. (2008) also reported that RWC declined in olive leaves after 5 days of water stress, which sank further by an average of 28% after 15 days of water stress. Gutierrez et al. (2010) observed declining RWC values in wheat genotypes with increasing drought stress. These results are in good agreement with those reported by Winterhalter et al. (2011), who found that maize cultivars subjected to drought stress exhibited greater decreases in water content throughout their different growth stages than they did under normal irrigation.

Regardless of the irrigation regime employed, no significant differences were observed between Koroneiki and T2 with respect to RWC, SWC, or spectral indices, neither did the interaction between irrigation regime and cultivar have any significant effects on these parameters. Compared with the control, however, these cultivars exhibited significant decreases in RWC, SWC, and WI but significant increases in their normalized spectral indices. Moreover, the leaf level WI and NWI1–5 values that are extensively used for RWC (Gutierrez et al., 2010; Penuelas et al., 1993; Serrano et al., 2000) and SWC (Gutierrez et al., 2010) assessments showed significant changes in plants subjected to drought stress (Table 1).

Spectral indices were strongly affected by irrigation regimes in July and November and when averaged over the three measurements, while no significant differences in spectral indices were observed between irrigation treatments in December although the five normalized spectral indices and WI were slightly higher in the deficit plants and the control, respectively. Indeed, these five normalized spectral indices increased in the two olive cultivars studied while WI decreased with increasing water stress. For example, as reported in Table 1, the mean values obtained from the three measurements ranged from −.0194 to −.0174 for the normalized water stress index‐4 (R970 − R880)/(R970 + R880) and from 1.0425 to 1.0366 for the WI (R970/R900).

These results are in good agreement with those observed by Gutierrez et al. (2010), who reported that, compared to water‐stressed conditions, advanced lines of spring wheat cultivars recorded the lowest values of NWI‐1 (R970 − R900)/(R970 + R900); NWI‐2 (R970 − R850)/(R970 + R850); NWI‐3 (R970 − R920)/(R970 + R920); and NWI‐4 (R970 − R880)/(R970 + R880) under good irrigation. While Marino et al. (2014) found that PRI, NDVI, and WI were affected by drought and recorded significantly higher values in the control olive trees than in the rainfed ones, Sun et al. (2008) reported no effect of water stress on WI.

### 3.3 Relationships of spectral indices with RWC and SWC

**FIGURE 3:**
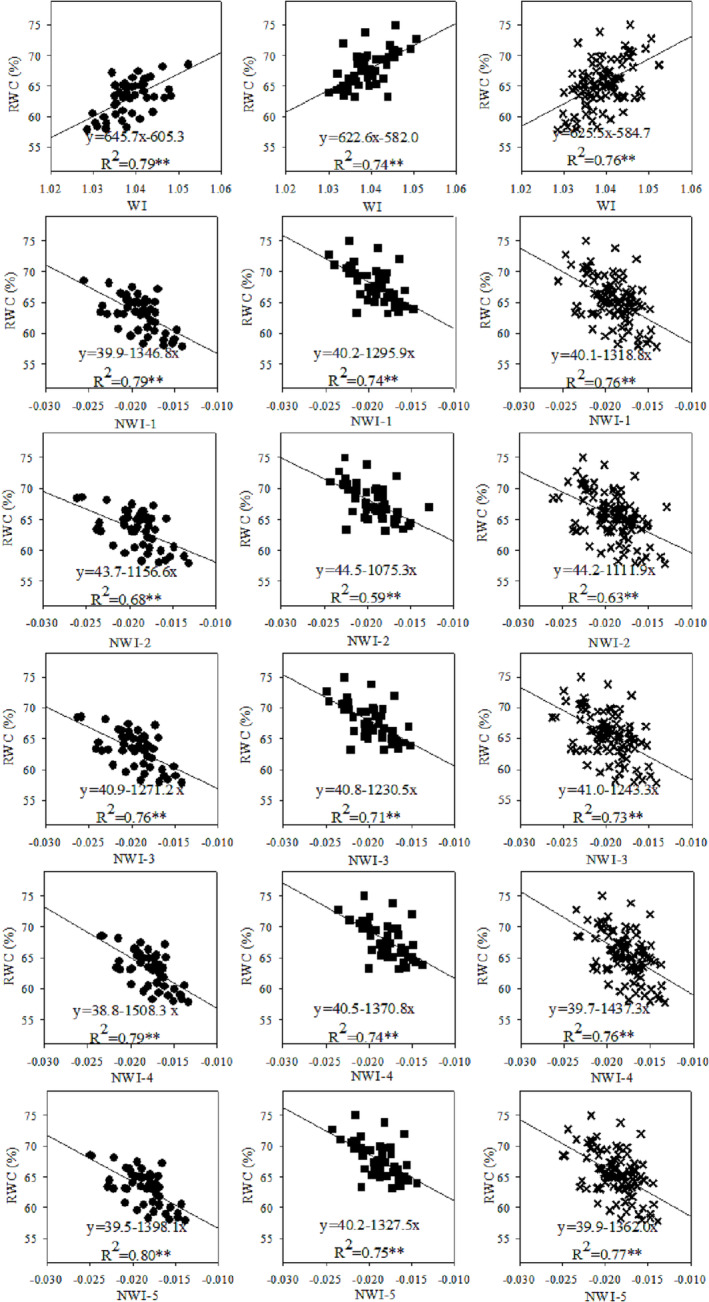
Relationships between spectral indices (WI and NWI1–5) and RWC in Kroneiki (•), T_2_ (∎), and both cultivars (×) subjected to the four irrigation regimes in the three measurements during the growing season with linear regressions fitted. *Statistically significant at *p* ≤ .05 and **statistically significant at *p* ≤ .01, respectively.

**FIGURE 4:**
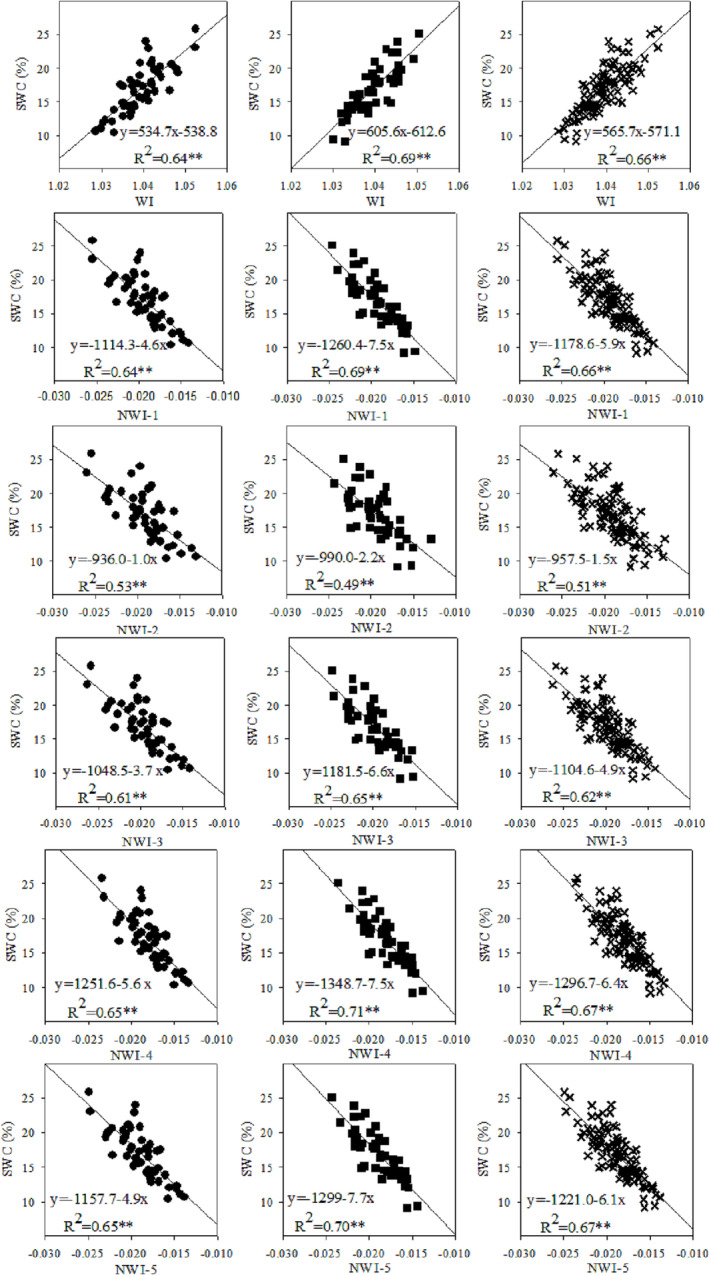
Relationships between spectral indices (WI and NWI1–5) and SWC in Kroneiki (•), T_2_ (∎), and both cultivars (×) subjected to the four irrigation regimes in the three measurements during the growing season with linear regressions fitted. *Statistically significant at *p* ≤ .05 and **statistically significant at *p* ≤ .01, respectively.

The selected spectral indices were found in closely related to RWC and SWC not only for each cultivar and measurement but across the two olive cultivars as well (Table 2). The coefficients of determination (*R* ^2^) obtained are reported in Table 2 and in Figures 3 and 4. The statistically significant linear relationships between the selected spectral indices derived from the near‐infrared region varied for both RWC (*R* ^2^ = .5, *p* ≤ .01 to .82, *p* ≤ .01) and SWC (*R* ^2^ = .26, *p* ≤ .05 to .77, *p* ≤ .01) recorded for the olive cultivars. Their relationships were affected by measurement time (Table 2), probably due to changes in environmental conditions.

**TABLE 2:** Coefficients of determination (*R* ^2^), slope (a), and intercept (b) of the relationships established for spectral indices with RWC and SWC in Koroneiki, T2, and both cultivars subjected to the four irrigation regimes in the first, second, and third rounds of measurements.

Olive cultivar	Spectral indices	July 3						October 29						December 16					
		RWC			SWC			RWC			SWC			RWC			SWC		
		a	b	$R^{2}$	a	b	$R^{2}$	A	b	$R^{2}$	a	b	$R^{2}$	a	b	$R^{2}$	a	b	$R^{2}$
Koroneiki	WI	598.8	−557.7	.70**	388.8	−388.5	.54**	383.4	−332.9	.72**	567.2	−572.7	.57**	496.3	−448.3	.77**	322.1	−316.1	.36*
	NWI−1	−1242.8	40.8	.70**	−806.8	−.1	.54**	−803.0	50.1	.72**	−1189.0	−6.1	.57**	−1037.2	47.5	.77**	−673.2	−5.8	.36*
	NWI−2	−1090.3	43.7	.59**	−667.3	2.5	.41**	−613.6	53.5	.64**	−953.8	−1.9	.56**	−905.2	50.8	.82**	−505.8	9.6	.29*
	NWI−3	−1170.1	41.7	.65**	−741.8	.8	.48**	−717.9	51.3	.7**	−1088.0	−4.7	.58**	−990.9	48.2	.80**	−616.3	6.8	.34*
	NWI−4	−1304.4	41.1	.71**	−840.9	.2	.54**	−936.9	48.7	.74**	−1359.3	−7.6	.56**	−1197.6	46.0	.76**	−833.3	3.7	.41**
	NWI−5	−1269.2	40.8	.71**	−826.9	.2	.56**	−860.5	49.4	.73**	−1260.6	−6.9	.57**	−1085.3	47.0	.76**	−722.1	5.0	.38*
T2	WI	431.1	−384.8	.64**	350.0	−349.2	.52**	292.9	−264.7	.79**	362.2	−360.0	.76**	439.5	−445.7	.77**	430.9	−428.8	.53**
	NWI−1	−895.0	46.0	.64**	−727.3	.6	.52**	−610.5	52.3	.78**	−754.1	1.9	.76**	−1031.0	47.2	.77**	−899.9	1.7	.53**
	NWI−2	−607.9	51.1	.50**	−435.7	5.8	.32**	−571.1	53.2	.72**	−695.0	2.9	.70**	−840.7	51.5	.60**	−585.3	8.5	.26*
	NWI−3	−790.2	47.5	.59**	−626.2	2.1	.46**	−596.0	52.3	.77**	−727.4	2.0	.74**	−1003.3	47.4	.76**	−833.4	2.7	.47**
	NWI−4	−923.0	46.6	.62**	−758.6	.90	.52**	−657.9	52.5	.80**	−805.0	2.1	.77**	−1101.7	47.3	.80**	−1023.0	.5	.61**
	NWI−5	−919.5	45.9	.64**	−752.3	.50	.53**	−624.7	52.4	.79**	−771.9	2.0	.77**	−1059.9	47.1	.78**	−952.4	1.0	.57**
Both cultivars	WI	523.3	−480.0	.65**	368.2	−367.6	.52**	258.0	−289.0	.72**	452.1	−453.2	.58**	495.3	−447.4	.77**	−357.3	362.0	.41**
	NWI−1	−1086.4	43.0	.65**	−764.5	.30	.52**	−536.1	51.5	.71**	−944.0	−1.6	.58**	−1035.0	47.54	.77**	−765.4	4.5	.41**
	NWI−2	−818.9	47.9	.50**	−532.2	4.4	.34**	−485.7	53.50	.66**	−828.9	.40	.58**	−878.9	51.0	.72**	−537.0	9.2	.27**
	NWI−3	−980.7	44.50	.59**	−675.7	1.6	.45**	−507.2	52.1	.70**	−893.3	−1.1	.59**	−989.7	48.0	.77**	−697.5	5.4	.39**
	NWI−4	−1137.2	43.4	.64**	−799.9	.6	.51**	−587.6	51.1	.73**	−1033.6	−1.9	.57**	−115.5	46.6	.76**	−919.1	2.3	.49**
	NWI−5	−1115.4	43.0	.66**	−787.8	.2	.53**	−555.6	51.3	.72**	−979.5	−1.8	.58**	−1076.1	47.0	.76**	−809.0	3.6	.44**

* Statistically significant at *p* ≤ .05.

** Statistically significant at *p* ≤ .01.

Generally, the normalized spectral water index of NWI‐2 (i.e., (R970 − R850)/(R970 + R850)) showed the least relationship with RWC and SWC across the measurements (lower in the range 1–35% when compared with the other spectral indices). The four spectral indices of WI, NWI‐1, NWI‐4, and NWI‐5 sometimes demonstrated similar results without significant differences among them. Moreover, these four spectral indices were found more accurate than the two NWI‐3 and NWI‐2 in predicting the RWC and SWC in olive trees.

A growing body of studies shows significant correlations between different water related parameters and spectral indices at different wavelengths, suggesting non‐destructive and expeditious spectroradiometric methods of plant water status assessment (El‐Hendawy, Al‐Suhaibani, et al., 2017; Elsayed et al., 2017; Erdle et al., 2011, 2013). This is line with the report by Serrano et al. (2000), who showed that both NIR‐based NDVI and WI serve as good indicators of Chaparral vegetation water status. Eitel et al. (2006) also reported statistically significant correlations between plant water status parameters (viz., RWC, soil water potential [SWP], LWP, and equivalent water thickness [EWT]) and the four spectral indices of WI, NDWI, the red edge inflection point, and the maximum difference water index: (Rmax 1,500–1,750 –Rmin 1,500–1,750)/(Rmax 1,500–1,750 +Rmin 1,500–1,750) derived from spectra taken at the leaf level of Populus spp.

Gutierrez et al. (2010) reported strong associations between the five canopy spectral indices of WI and NWI1–4 and plant and soil water parameters under field water stress condition. Elsayed et al. (2017) showed that the different water related parameters (i.e., RWC and CWC), and grain yield (GY) in wheat cultivars were significantly correlated with NWI‐1, NWI‐3, NWI‐4, NWI‐5, and NWI‐990‐992. However, the present results are in contrast with those reported by Sun et al. (2008), who estimated WI on detached leaves of water‐stressed olive saplings grown in pots. It is suggested that experimental design and measurement methodology might account for the conflicting results observed.

Pooling together the values collected from all the measurements led to the establishment of a significantly linear relationship (*R* ^2^ = .51 to .77, *p* < .01) between RWC and the five normalized spectral indices (viz., NWI‐1, NWI‐2, NWI‐3, NWI‐4, and NWI‐5) alongside WI (Figure 3). Furthermore, the spectral indices also scaled linearly with SWC (Figure 4) albeit these latter relationships were weaker than the former (i.e., *R* ^2^ = .45 to .67, *p* < .01).

### 3.4 RWC and SWC modeling

Regression curves were fitted to develop empirical models based on the linear relationships derived and using a dataset of 64 data from the first and second measurements (67% of each measured RWC and SWC) used as training data with the remaining 32 data from the third measurement (33%) retained as test data. The statistical parameters included coefficients of determination (*R* ^2^) and RMSE. Furthermore, the intercept and slope of the linear regressions between the observed and predicted values were calculated as a measure of the difference between observed and predicted values for RWC and SWC.

**FIGURE 5:**
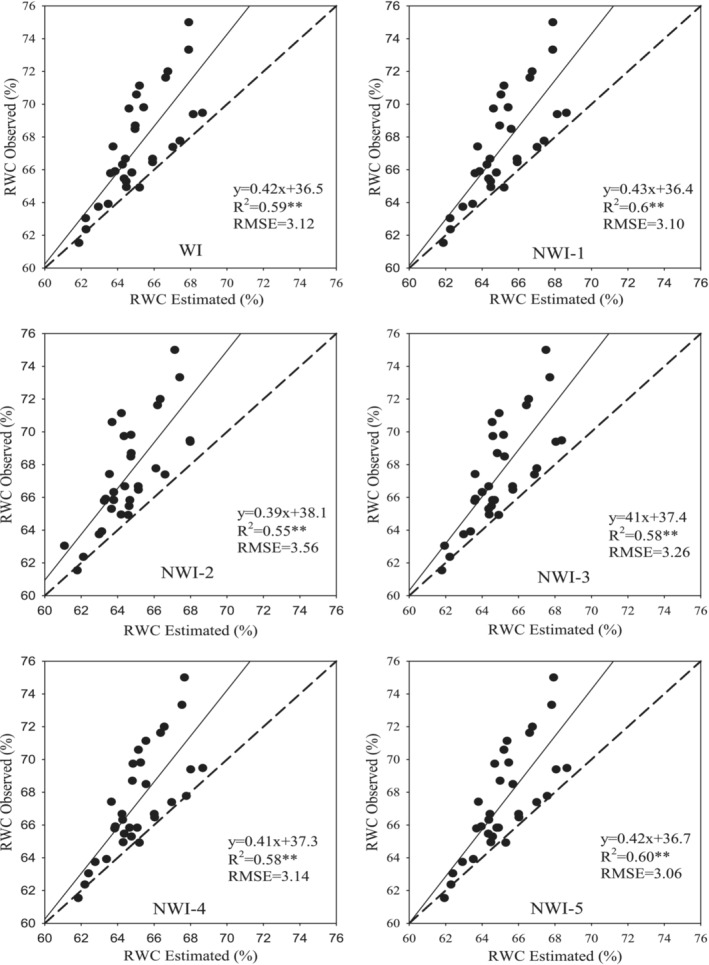
Scatter plots containing root‐mean‐square error (RMSE), coefficients of determination (*R* ^2^), and functions of linear relationships between observed and predicted values of relative water content based on the data of six spectral indices plotted on 1:1 line. The calibration functions were validated using independent data obtained from the third set of measurements.

**FIGURE 6:**
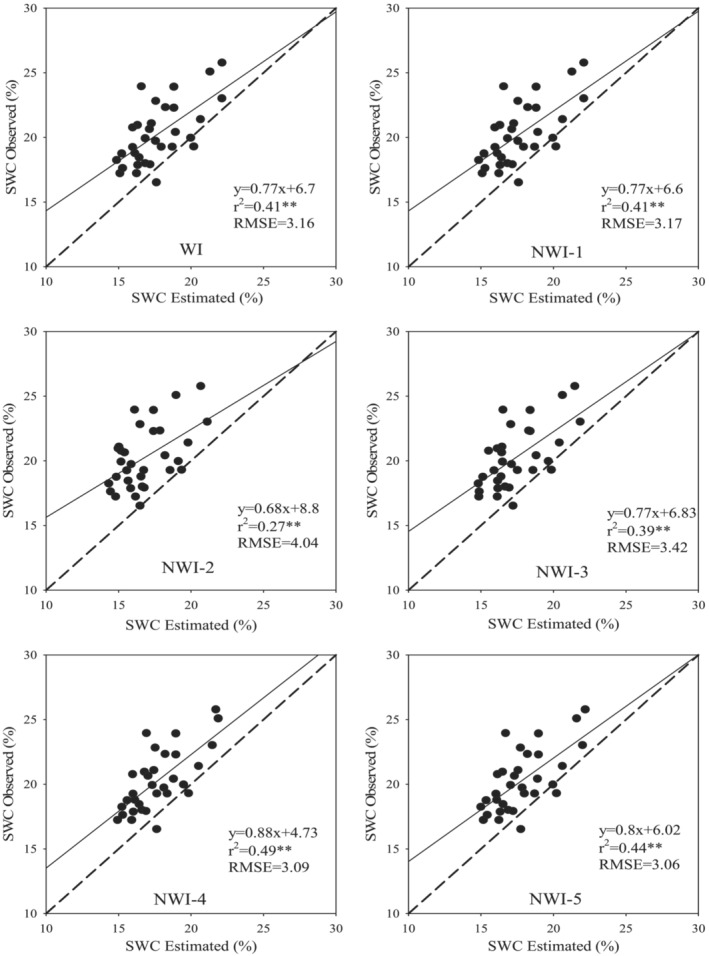
Scatter plots containing root‐mean‐square error (RMSE), coefficients of determination (*R* ^2^), and functions of linear relationships between observed and predicted values of soil water content (SWC) based on the data of six spectral indices plotted on 1:1 line. The calibration functions were validated using independent data obtained from the third set of measurements.

Comparisons of the parameters estimated using the empirical models and the actual measured data are presented in Figures 5 and 6 that simultaneously shows the various statistical parameters used for the validation of the models and determining their accuracy based on differences between the observed and predicted RWC and SWC values on a 1:1 scatter plot line. A model with the highest values of *R* ^2^ and slope but the lowest values of RMSE was identified as one with the highest predictive accuracy. The models that fulfilled most of the validation criteria based on the four statistical parameters were selected for accurate prediction of RWC (Figure 5) and SWC (Figure 6). Based on these criteria, the different models of the six spectral indices yielded more accurate estimates of RWC than they did of SWC. The results also revealed that the spectral indices studied were effective in estimating relative values of both olive leaf RWC and SWC as indicators of plant water stress. Among the six spectral indices, four satisfied most of the criteria used to determine the accuracy of the models in predicting RWC and SWC (Figures 5 and 6). It is clear that the models based on WI, NWI‐1, NWI‐4, and NWI‐5 provided more accurate estimates of RWC and SWC albeit the estimated values of the parameters studied were always less than the measured ones over the entire set of measurements. These results confirmed the ability of spectral indices for non‐destructive and rapid determination of RWC in olive leaves and SWC that provide a quantitative measure of the water status of the olive tree in the field, feasibility of optimal and precise management of irrigation, something that will have great importance to olive cultivation in arid countries such Iran.

## 4 CONCLUSION

Olive is becoming a strategic crop worldwide, and the olive orchards are being increasingly irrigated to enhance its production. To save irrigation water and increase its productivity, there is a pressing need for monitoring plant water status to detect signs and degrees of prevalent water stress. It was hypothesized that the RWC levels of olive cultivars and the SWCs of cultivation sites could be reliably determined using spectral indices under different irrigation regimes. The present study was, hence, conducted on mature olive plantations in an arid environment. The findings indicated that (a) RWC, SWC, and spectral indices of WI and NWI 1–5 estimated at the leaf level were significantly affected by irrigation regimes; (b) the above six indices showed strong linear associations with RWC and SWC; (c) WI, NWI‐1, NWI‐4, and NWI‐5 outperformed NWI‐3 and NWI‐2 in predicting RWC and SWC levels as plant water status indices in olive trees; and (d) spectral indices yielded more accurate estimates of RWC than they did of SWC. Overall, the study clearly showed that the spectral indices of WI and NWI 1–5 measured at the leaf level might serve as good stress indicators in olive trees and that they may be recommended as rapid and non‐destructive detectors of water status to achieve improved deficit irrigation management in arid regions.

## AUTHOR CONTRIBUTIONS

**Abdolrahim Hooshmand**: Supervision; funding acquisition; review and editing. **Azimeh Asgari**: Conceptualization and design of the experiment; data curation; formal analysis; writing, review and editing. **Saeed Broumand‐Nasab** and **Shohreh Zivdar**: Review and editing. All authors contributed significantly and read and approved the article.

## CONFLICT OF INTEREST STATEMENT

The authors declare that they have no conflict of interest.

### PEER REVIEW

The peer review history for this article is available in the Supporting Information for this article.

## Supporting information

DECISION LETTER (Round 1)

## Data Availability

The data that supporting the findings of this study are available from the corresponding author.
