# Supplementary material for: Potential application of spectral indices for olive water status assessment in (semi‐)arid regions: A case study in Khuzestan Province, Iran
Source: Plant Direct. 2023 Jun 6;7(6):e494. doi: 10.1002/pld3.494 (PMC10244469; doi:10.1002/pld3.494)
Supplement: Supplementary file 1 — DECISION LETTER (Round 1) [file PLD3-7-e494-s001.docx]

**DECISION LETTER (Round 1)**

January 3, 2023

Dr. Abdolrahim Hooshmand
Shahid chamran university of Ahvaz
Golestan highway
Ahvaz
Iran


RE: Potential application of spectral indices for olive water status assessment in (semi-)arid regions (A case study in Khuzestan Province, Iran)

Dear Dr. Hooshmand:

Thank you for submitting to Plant Direct. All required reviews have been returned and we have now finished our evaluation of your manuscript. In light of the reviewers' and editor's comments, further revisions are needed before the paper can be accepted for publication in Plant Direct.

Please view the editors' and reviewers' comments below and use their suggestions as a guide while you work on your revision.

When uploading the revised version of this article, please be sure to include the following:

-A word document that contains your response to the reviewers. You should respond to each reviewer comment and note the changes made to the manuscript. If you do not agree with a reviewer's comment and choose not to make a suggested revision, please explain why. Please try to provide as complete an answer as possible to each reviewer's criticisms.
-A tracked changes document with each change highlighted
- A clean version of the latest version of the manuscript


To upload your revision, please click the link below.
https://plantdirect.msubmit.net/cgi-bin/main.plex?el=A4Lr7XB7A6nlf7I5A9ftd2cGaXL3WXaFekzDxJ8dRAZ


In order to provide as timely a service as possible, we ask that your revision is resubmitted within three months after receipt of this request. If an extension is needed, please send a request, along with a brief explanation, to the editorial office at plantdirect@wiley.com .

Please note that, in addition to publishing reviewer comments, the author's responses to review comments will also be published alongside the final version of the paper. If you would not like the author's responses to be published, please contact the editorial office at plantdirect@wiley.com .


Thank you very much for giving us an opportunity to review your work. I look forward to receiving the next version.


Sincerely,

Larry York


Editor, Plant Direct


---------------------------------------------------------------------------- Editor comments:

Better method descriptions are required and additional multivariate analyses to make use of the data appropriately.


---------------------------------------------------------------------------- Reviewer comments:
Reviewer #1:

The manuscript of Asgari and coauthors report experimental results that aim to use spectral indices to assess relative water content (RWC) in olive trees as well as soil water content (SWC) under different irrigation conditions. The authors utilized two different cultivars from an orchard that contained 17-year-old olive trees that were treated with four different irrigation treatments. The authors collected spectral reflectance measurements with a portable spectrometer, collected RWC measurements in leaves, and SWC measurements. The authors used five previously published spectral indices for analysis and confirmed linear relationships with both RWC and SWC measurements. Furthermore, the authors presented empirical models for RWC and SWC estimation. The research demonstrates that these spectral indices may serve as indicators of water stress in olive orchards. Before publication, several points need to be address and the manuscript revised.

-Please provide the number of sampling and data collection dates. If data were collected on a single date only, the authors should increase the number of collection dates and data sets to validate and verify model performance over time and variable conditions.

-The authors should expand the discussion on the practical use of these spectral indices "...for monitoring plant water status to detect signs and degrees of prevalent water stress."...possibly for smart irrigation practices.

-A field map should be included as a main or supplemental figure with features of the experimental design.
-In the Materials and Methods section, please explain in detail the split plot design with RCBD.

-A One-Way ANOVA was used for analysis. However, the authors should consider using a Two-Way ANOVA for analysis to evaluate differences and similarities between two cultivars and four irrigation treatments.

-Please provide more information on how specific data sets were analyzed. Specifically, provide details for ANOVA analysis and mean separation ("subjected to analysis of variance (one-way ANOVA) and the least significant difference (LSD) test was used to separate the means (p{less than or equal to}0.05 and p{less than or equal to}0.01).") Please report p values for each ANOVA performed.

-Please clearly state the total number of trees used in the study as well as the number of trees included for each cultivar.

-For sampling, please provide the number of leaves collected from each tree and how the leaves were randomly chosen and collected.

-Please indicate if spectral reflectance measurements were taken on the same leaves used to measure relative water content.

-Table 1. Standard deviation or standard error should be included.

-Figures 2 and 3: Please adjust the location of text in panels so that it is not obscured by data points. For clarity, please label panels or panel groups with the names of spectral indices.

Reviewer #2:

Even with the intensive multivariate data, the manuscript lacks multivariate statistical methods. For example, while separation of genotypes failed with univariate methods, multivariate methods like MANOVA or Linear Discriminant Analysis may do a better job.

In terms of regression, it would be sensible to see if multiple wavelengths and interactions can predict water content. The authors could try stepwise regression and random forest, or more advanced machine learning.

Inclusion of these multivariate methods and a better description of methods would greatly improve the manuscript. A figure 1 with overhead images of the orchard and methods used would be useful.

**DECISION LETTER (Round 2)**

March 15, 2023

Dr. Abdolrahim Hooshmand
Shahid chamran university of Ahvaz
Golestan highway
Ahvaz
Iran


RE: Potential application of spectral indices for olive water status assessment in (semi-)arid regions (A case study in Khuzestan Province, Iran)

Dear Dr. Hooshmand:

Thank you for submitting to Plant Direct. All required reviews have been returned and we have now finished our evaluation of your manuscript. In light of the reviewers' and editor's comments, further revisions are needed before the paper can be accepted for publication in Plant Direct. If the revisions can be made quickly, I expect to make an accept decision once receiving the revised version.

Please view reviewers' comments below and use their suggestions as a guide while you work on your revision.

When uploading the revised version of this article, please be sure to include the following:

-A word document that contains your response to the reviewers. You should respond to each reviewer comment and note the changes made to the manuscript. If you do not agree with a reviewer's comment and choose not to make a suggested revision, please explain why. Please try to provide as complete an answer as possible to each reviewer's criticisms.
-A tracked changes document with each change highlighted
- A clean version of the latest version of the manuscript


To upload your revision, please click the link below.
https://plantdirect.msubmit.net/cgi-bin/main.plex?el=A4Lr2XB2B6nlf7I1A9ftd2cGaXL3WXaFekzDxJ8dRAZ


In order to provide as timely a service as possible, we ask that your revision is resubmitted within three months after receipt of this request. If an extension is needed, please send a request, along with a brief explanation, to the editorial office at plantdirect@wiley.com .

Please note that, in addition to publishing reviewer comments, the author's responses to review comments will also be published alongside the final version of the paper. If you would not like the author's responses to be published, please contact the editorial office at plantdirect@wiley.com .


Thank you very much for giving us an opportunity to review your work. I look forward to receiving the next version.


Sincerely,

Larry York

Editor, Plant Direct


---------------------------------------------------------------------------- Reviewer comments:
Reviewer #1:

The revised manuscript submitted by Asgari and coauthors is improved and has addressed several specific comments from the previous submission. The experiments and results reported by the authors meet the technical standards for experimental design and data analysis. Also, the reported results support the authors conclusions. The manuscript describes the experiments and data analysis in adequate detail with two exceptions. Before publication, the following minor points should be addressed.

1. Details for multivariate analysis should be included in the Materials and Methods section, 2.6. Statistical analysis, lines 214-215.
2. Line 528 and the Figure 1 legend should be edited to include the terms split-plot and RCBD.

**AUTHOR RESPONSE**

**Point-by-point response to the comments of the reviewers and/or editors**

We thank the referees for expressing interest in our study and for their insightful comments that improved our manuscript.

**Editor**

- Better method descriptions are required and additional multivariate analyses to make use of the data appropriately.

*Thank you for your feedback. For better method description:*

*-* *A map of experimental design has been added in material and methods section (figure 1).*

*-* *The numbers of sampling and data collection dates has been added in material and methods section (lines 162-163, 176-177 and 179-181).*

*-Total numbers of trees used in this study as well as the numbers of trees included for each cultivar, has been clarified in material and methods section (lines 133-134).*

*- the numbers of leaves collected from each tree and how the leaves were chosen and collected, has been added in material and methods section (lines 161-167).*

*And multivariate analysis has been done (lines 208-209).*

**Revisions required for publication**

**Reviewer #1**

The manuscript of Asgari and coauthors report experimental results that aim to use spectral indices to assess relative water content (RWC) in olive trees as well as soil water content (SWC) under different irrigation conditions. The authors utilized two different cultivars from an orchard that contained 17-year-old olive trees that were treated with four different irrigation treatments. The authors collected spectral reflectance measurements with a portable spectrometer, collected RWC measurements in leaves, and SWC measurements. The authors used five previously published spectral indices for analysis and confirmed linear relationships with both RWC and SWC measurements. Furthermore, the authors presented empirical models for RWC and SWC estimation. The research demonstrates that these spectral indices may serve as indicators of water stress in olive orchards. Before publication, several points need to be address and the manuscript revised.

1-Please provide the number of sampling and data collection dates. If data were collected on a single date only, the authors should increase the number of collection dates and data sets to validate and verify model performance over time and variable conditions**.**

*We thank the reviewer for pointing out this omission. Relative water content (RWC) in olive trees, soil water content (SWC) as well as spectral measurements were carried out three times under clear sky condition, on July 3, October 29 and December 16, 2018.* *This has been clarified in the material and methods section (lines 162-163, 176-177 and 179-181).*

2-The authors should expand the discussion on the practical use of these spectral indices "...for monitoring plant water status to detect signs and degrees of prevalent water stress."...possibly for smart irrigation practices.

*This study sought to ascertain the ability of several spectral indices including water index (WI) and normalized spectral water indices 1-5 (NWI 1-5) to estimate water status in olive trees in arid regions in Iran. The results confirmed the ability of spectral indices for non destructive and rapid determination of RWC in olive leaves and SWC that provide a quantitative measure of the water status of the olive tree in the field, enabling optimal and precise management of irrigation, something that will have great importance to olive cultivation in arid countries such Iran. This has been clarified in results and discussion section (lines 338-341).*

3-A field map should be included as a main or supplemental figure with features of the experimental design.

*As suggested by the reviewer, a map of experimental design was added in material and methods section, figure 1 and figures number has been updated.*

4-In the Materials and Methods section, please explain in detail the split plot design with RCBD.

*This study was performed on 17-year-old mature olive trees of ‘Koroneiki’and ‘T_2_’ cultivars. The experimental design was split plot, arranged based on Randomized Complete Block Design (RCBD) with four replications. The two olive cultivars were designed as the main factors and the four irrigation regimes as the sub-main factors. It has been clarified in material and methods section (lines 131-133). For better description, a map of experimental design has been added (figure 1).*

5-A One-Way ANOVA was used for analysis. However, the authors should consider using a Two-Way ANOVA for analysis to evaluate differences and similarities between two cultivars and four irrigation treatments.

*As suggested by the reviewer MANOVA was done* *and lines 208-209 in materials and methods section have been corrected as "Results were subjected to analysis of variance (ANOVA) based on split plot design and the least significant difference (LSD) test was used to separate the means (p≤0.05)."*

6-Please provide more information on how specific data sets were analyzed. Specifically, provide details for ANOVA analysis and mean separation ("subjected to analysis of variance (one-way ANOVA) and the least significant difference (LSD) test was used to separate the means (p{less than or equal to}0.05 and p{less than or equal to}0.01).") Please report p values for each ANOVA performed.

*Analysis of variance (ANOVA) based on split plot design was conducted on the relative water content, soil water content and spectral indices using SAS software (version 9, SAS Institute, 2001), and significance of differences among treatments were separated using Least Significant Difference (LSD) at a 5% probability level and lines 208-209 in materials and methods section have been corrected as "Results were subjected to analysis of variance (ANOVA) based on split plot design and the least significant difference (LSD) test was used to separate the means (p≤0.05).*

7-Please clearly state the total number of trees used in the study as well as the number of trees included for each cultivar.

*A total of 32 trees were used in this study* *(16 trees of each cultivar). Such that four trees (one tree per block) of each cultivar per irrigation treatment could be measured. This has been clarified in materials and methods section (lines 133-134).*

8-For sampling, please provide the number of leaves collected from each tree and how the leaves were randomly chosen and collected.

*The leaf sampling was accomplished on three different days on July 3, October 29 and December 16, 2018 at midday between 11.00 h and 14.00 h under cloudless conditions. In overall in every single sampling, eight fully-developed sun-exposed leaves, which were located in the middle of canopy were excised from each tree (five leaves were used for Relative water content (RWC) measurements and three more leaves were used for spectral measurements). The leaf samples were then sealed in plastic bags and kept at temperatures below 5* $℃$ *before they were transferred to the laboratory. This has been clarified in materials and methods section (lines 161-167).*

9-Please indicate if spectral reflectance measurements were taken on the same leaves used to measure relative water content.

*Spectral reflectance measurements were performed on leaf samples separate from leaf samples used to measure relative water content.*

*The leaf sampling was accomplished on three different days on July 3, October 29 and December 16, 2018 at midday between 11.00 h and 14.00 h under cloudless conditions. In overall in every single sampling, eight fully-developed sun-exposed leaves, which were located in the middle of canopy were excised from each tree (five leaves were used for Relative water content (RWC) measurements and three more leaves were used for spectral measurements). The leaf samples were then sealed in plastic bags and kept at temperatures below 5* $℃$ *before they were transferred to the laboratory. This has been clarified in materials and methods section (lines 161-167).*

10-Table 1. Standard deviation or standard error should be included.

*As suggested by the reviewer, standard deviation has been included in table 1 (Tables section, page 11).*

11-Figures 2 and 3: Please adjust the location of text in panels so that it is not obscured by data points. For clarity, please label panels or panel groups with the names of spectral indices.

*As suggested by the reviewer, figures 2 and 3 have been corrected.*

**Reviewer #2**

Even with the intensive multivariate data, the manuscript lacks multivariate statistical methods. For example, while separation of genotypes failed with univariate methods, multivariate methods like MANOVA or Linear Discriminant Analysis may do a better job.

*Thank you for your feedback. MANOVA was done and lines 208-209 have been corrected as "Results were subjected to analysis of variance (ANOVA) based on split plot design and the least significant difference (LSD) test was used to separate the means (p≤0.05)".*

In terms of regression, it would be sensible to see if multiple wavelengths and interactions can predict water content. The authors could try stepwise regression and random forest, or more advanced machine learning.

*To adress the research question we used simple linear regressions to analyze the relationships of spectral reflectance indices with relative water content and soil water content and to determine coefficients of determination. We will consider your point for future studies.*

Inclusion of these multivariate methods and a better description of methods would greatly improve the manuscript. A figure 1 with overhead images of the orchard and methods used would be useful.

*As suggested by the reviewer, a map of experimental design was added in material and methods section, figure 1 and figures number has been updated.*

*MANOVA was done and lines 208-209 have been corrected as "Results were subjected to analysis of variance (ANOVA) based on split plot design and the least significant difference (LSD) test was used to separate the means (p≤0.05)".*

**DECISION LETTER (Round 3)**

March 27, 2023
Dr. Abdolrahim Hooshmand
Shahid chamran university of Ahvaz
Golestan highway
Ahvaz
Iran


MSID: 2022-01014R2
MS TITLE: Potential application of spectral indices for olive water status assessment in (semi-)arid regions (A case study in Khuzestan Province, Iran)

Dear Dr. Abdolrahim Hooshmand:

I am pleased to inform you that your manuscript "Potential application of spectral indices for olive water status assessment in (semi-)arid regions (A case study in Khuzestan Province, Iran)" has been accepted for publication in Plant Direct.

Your article will appear online in the next available issue of Plant Direct. To ensure your article gets published as quickly as possible, please pay attention to the steps detailed below. We have found that most of the delays happen at this stage, especially at the payment stage, so please respond as quickly as possible when prompted.

License Agreement: Once your article has been accepted it will move to Production and undergo admin and file checking - you may receive an email with any queries we have at this stage. When all required items are received by the publisher and queries resolved, the corresponding author will receive an email from Wiley's Author Services system which will ask them to log on at https://authorservices.wiley.com/bauthor and will present them with the appropriate license for completion. Your article cannot be published until both the signed license agreement and payment of the article fee have been received.

Payment of the Open Access Article Publication Fee: All articles published in Plant Direct are fully open access: immediately and freely available to read, download and share. Plant Direct charges a publication fee to cover the publication costs. The corresponding author for this manuscript should have already received a quote from the payments team (cs-openaccess@wiley.com) with the estimated article publication fee; please email cs-openaccess@wiley.com if this has not been received. The corresponding author should log on to the Wiley Author Services site, where the publication fee can be paid by credit card or an invoice. Pro Forma can also be requested. Payment of the publication charge must be received before the article will be published online.

Proofs: You will have the opportunity to look over your paper once more when you receive the author proofs for your article. The proofs will be with you in approximately two weeks. Please note that, in addition to publishing reviewer comments, the author's responses to review comments will also be published alongside the final version of the paper. If you would not like the author's responses to be published, please contact the editorial office at plantdirect@wiley.com .

Promotion of your article: You can help your research get the attention it deserves! Check out Wiley's free Promotion Guide for best-practice recommendations for promoting your work at www.wileyauthors.com/eeo/guide. And learn more about Wiley Editing Services which offers professional video, design, and writing services to create shareable video abstracts, infographics, conference posters, lay summaries, and research news stories for your research at www.wileyauthors.com/eeo/promotion.

Thank you again for your contribution to Plant Direct. If you have any questions, feel free to contact the editorial office at plantdirect@wiley.com .

Sincerely,

Larry York

Editor, Plant Direct

**AUTHOR RESPONSE**

Point-by-point response to the comments of the reviewers and/or editors

We thank the referees for expressing interest in our study and for their insightful comments that improved our manuscript

---------------------------------------------------------------------------- Reviewer comments:
Reviewer #1:

The revised manuscript submitted by Asgari and coauthors is improved and has addressed several specific comments from the previous submission. The experiments and results reported by the authors meet the technical standards for experimental design and data analysis. Also, the reported results support the authors conclusions. The manuscript describes the experiments and data analysis in adequate detail with two exceptions. Before publication, the following minor points should be addressed.

1. Details for multivariate analysis should be included in the Materials and Methods section, 2.6. Statistical analysis, lines 214-215.

*ANOVA uses when the response variable is continuous, and the factors of interest are categorical. The factors are also known as independent variables or predictors. In this study, the response variables were the soil water content (SWC), relative water content (RWC) and spectral indices and the factors were two olive cultivars and four irrigation regimes.*

*In a split-plot design, there are two or more factors, and the experimental units are divided into subunits. The factors are applied to the subunits, and the response variable is measured on each subunit. This type of design is used when the factors cannot be applied to the whole experimental unit at once or when there is a natural hierarchy of factors.*

*The least significant difference (LSD) test compares the difference between two means to the standard error of the difference, and if the difference is larger than a critical value, then the means are significantly different at the specified alpha level.*

*Analysis of variance (ANOVA) based on split plot design was conducted on the relative water content, soil water content and spectral indices using PROC GLM (General Linear Model) procedure of SAS software (version 9, SAS Institute, 2001), and significance of differences among treatments were separated using Least Significant Difference (LSD) at a 5% probability level and lines 208-209 in materials and methods section have been corrected as "* The ability of six spectral indices in assessing such water status related parameters in two olive cultivars as relative water content (RWC) and soil water content (SWC) under different irrigation regimes was investigated. The experiment was set up in a split plot design arranged based on the Randomized Complete Block Design (RCBD) with four replicates. The main factors consist of two olive cultivars and four irrigation regimes were tested as the sub-main factors. Analysis of variance (ANOVA) based on split plot design was conducted on the spectral indices, relative water content and soil water content using PROC GLM (General Linear Model) procedure of SAS software v.9.0 (SAS Institute, 2001). then, the least significant difference (LSD) test was used to compare the means and determine if they were significantly different from each other at the p≤0.05 level. SigmaPlot 10.0 software (Systat, San Jose, CA, USA) was used to create graphical representation of the data.*"*

2. Line 528 and the Figure 1 legend should be edited to include the terms split-plot and RCBD.

*As suggested by the reviewer, legend of figure 1 has been changed as "* Fig. 1. Experimental design layout of the field experiment in a split plot arrangement in a Randomized Complete Block Design (RCBD).**"**
